# Cell wall composition and biomass saccharification potential of *Sida hermaphrodita* differ between genetically distant accessions

**DOI:** 10.3389/fpls.2023.1191249

**Published:** 2023-06-29

**Authors:** Silvia D. Schrey, Jimena Martinez Diaz, Lukas Becker, Jane A. Mademann, Benedict Ohrem, Dagmar Drobietz, Pavel Chaloupsky, Nicolai D. Jablonowski, Christian Wever, Philipp M. Grande, Elena Pestsova, Holger Klose

**Affiliations:** ^1^ Institute of Bio- and Geosciences/Plant Sciences (IBG-2), Forschungszentrum Jülich GmbH, Jülich, Germany; ^2^ RWTH Aachen University, Aachen, Germany; ^3^ Institute of Developmental and Molecular Biology of Plants, Heinrich-Heine Universität Düsseldorf, Düsseldorf, Germany; ^4^ Department of Chemistry and Biochemistry, Mendel University in Brno, Brno, Czechia

**Keywords:** Virginia mallow, genotyping by sequencing, lignocellulose characterization, cell wall recalcitrance, OrganoCat

## Abstract

Due to its ample production of lignocellulosic biomass, *Sida hermaphrodita* (Sida), a perennial forb, is considered a valuable raw material for biorefinery processes. The recalcitrant nature of Sida lignocellulosic biomass towards pretreatment and fractionation processes has previously been studied. However, Sida is a non-domesticated species and here we aimed at expanding the potential of such plants in terms of their processability for downstream processes by making use of the natural variety of Sida. To achieve this goal, we established a collection comprising 16 different Sida accessions obtained from North America and Europe. First, we asked whether their cell wall characteristics are reflected in genetic distance or geographical distribution, respectively. A genotyping-by-sequencing (GBS) analysis resulting in a phylogenic tree based on 751 Single Nucleotide Polymorphisms (SNPs), revealed a high genetic diversity and a clear separation between accessions collected in North America and Europe. Further, all three North American accessions were separated from each other. Of the eleven European accessions, five form individual groups and six others belong to a single group. Clonal plants of seven selected accessions of American and European origin were produced and cultivated under greenhouse conditions and the resulting plant material was used for in-depth wet-chemical and spectroscopic cell wall characterization. Two accessions with contrasting cell wall characteristics were then selected and processed using the OrganoCat technology. Results of the different product yields and chemical compositions are reported. Overall, cell wall analyses revealed contrasting clusters regarding these main components between the accessions that can be related to genetic and, partly, geographical distance. Phenotypically, the accessions clustered into two groups that are not entirely overlapping with geographical origin. These results can be the basis for a targeted selection or cultivation of Sida accessions for biorefinery approaches.

## Introduction

Plant cell walls are the most abundant non-fossil carbon resource on earth (Bar-On et al., 2018). In the effort to develop (bio)chemical alternatives that could substitute petroleum-based chemicals and materials, the full utilization of plant cell walls is an important step (Jäger and Büchs, 2012).

Chemically, plant cell walls are composed of three main biopolymers, cellulose, hemicellulose, and lignin, with pectins and minor contributions from proteins and possibly lipids. One obstacle to the full valorization of lignocellulose is its recalcitrance to conversion into its building blocks which is a direct consequence of the complex nature of the cell wall structure (McCann and Carpita, 2015). In fully developed cell walls of higher plants, cellulose, a crystalline polysaccharide composed of glucose, is linked to lignin, a polyaromatic amorphous compound, via the amorphous polysaccharide hemicellulose, which varies in its composition of hexoses and pentoses (Harris and Stone, 2009). This complex may differ between plant species, developmental stages, and depending on environmental conditions during plant growth. As a consequence, approaches for the biomass component fractionation, chemical or enzymatic conversion of the lignocellulose, require adaptation, and, in turn, the chemicals and processes chosen may alter the quantity as well as the quality of the compounds obtained (McCann and Carpita, 2015). A variety of biomass conversion technologies, such as steam explosion (Jacquet et al., 2015), hydrothermal (Peterson et al., 2008), alkali (Kim et al., 2016; Li and Takkellapati, 2018), and acid treatments (Zhang et al., 2016) have been developed to overcome recalcitrance and facilitate subsequent conversion steps. Among them, methods like the OrganoCat process that aim to obtain structurally intact lignin while minimizing sugar degradation stand out. It uses a biphasic system comprising a biogenic solvent - to extract lignin *in situ* - and a catalyst - to hydrolyze non-cellulosic polysaccharides - under relatively mild conditions and allows straightforward recycling of water, solvent, and catalyst (Grande et al., 2015; Weidener et al., 2018; Weidener et al., 2021).

The drawback of using plant material for large-scale chemical industry lies in possible land use conflicts and competition with food production. To avoid such conflicts, plant material could either come from agricultural residues (e.g. Martinez Diaz et al., 2023) or be grown on land that is unsuitable for food or fodder production. Plants suitable for such an approach include perennial herbs and grasses that have significantly lower environmental impacts than annual crops and a higher range of ecosystem services (Clifton-Brown et al., 2023). Prominent and partly widespread examples are miscanthus (*Miscanthus* x *giganteus* Greef et Deuter), giant reed (*Arundo donax* L.), switchgrass *Panicum virgatum* (L.), and cup plant (*Silphium perfoliatum* L.).

At present, *Sida hermaphrodita* (L.) Rusby (called “Sida” here) is not widely cultivated. Sida, a perennial herbaceous plant in the mallow family (Malvaceae), also known as *Ripariosida hermaphrodita* (L.) Weakley & B.D. Poind, or by its common names Virginia fanpetals or Virginia mallow, originates in riparian landscapes of eastern North America, from where it was introduced to Poland and the former USSR in the 1930s, initially as a fodder and fiber crop (Cumplido-Marin et al., 2020). The advantages of Sida are numerous: the plants grow in light, sandy soils that are low in organic matter and even thrive on disturbed soils (Thomas, 1979). Sida produces yields of between 10 and 20 t ha^-1^ per year (Borkowska et al., 2009; Borkowska and Molas, 2013) while yields of up to 25 and 28 t ha^-1^ have been reported (Jablonowski et al., 2017; Nabel et al., 2017). At the same time, Sida is of high ecological value, e.g. for earthworms (Emmerling, 2014) and, due to their long flowering phase, for pollinators and honey production (Borkowska et al., 2006). In Europe, Sida has mostly been studied focusing on biomass production towards bioenergy generation (Borkowska and Molas, 2012; von Gehren et al., 2019; Jablonowski et al., 2020), and regarding management strategies for sustainable production on non-arable and marginal sites (Nabel et al., 2017; Nabel et al,, 2018). While Sida is a valuable crop for a variety of ecosystem services and can produce significant amounts of biomass even under less favourable conditions (Nahm and Morhart, 2018), it has recently been reported that the profitability of Sida as a bioenergy crop is negative, at least in Central Europe (Cumplido-Marin et al., 2022).

In previous works, Damm et al. (2017a); Damm et al. 2017b); Weidener et al. (2020), gained insight into the structural composition of Sida lignocellulose. To understand the non-cellulosic polysaccharide composition and its saccharification efficiency to enzymatic hydrolysis, the authors applied multiple analytical techniques, including OrganoCat, and identified pectic polysaccharides and xylans associated with lignin as responsible for Sida cell wall recalcitrance.

However, there is no specific Sida genotype that is used commercially or that has been described in detail. This makes it difficult to compare studies regarding e.g. biomass production, soil preferences, or disease susceptibilities, because the used germplasms may be different and associated traits may vary. Knowing whether a desired trait is due to genotype or a result of environmental conditions, or both is essential, especially when aiming to valorize the plant material for potential high-value compounds. Thus, there may be unexploited genetic diversity in natural populations of Sida genotypes that have adapted to different geographic conditions, including following their introduction to Europe. Such genetic diversity may affect not only environmental preferences or biomass production but also cell wall traits.

In our endeavor to elucidate the genetic diversity of Sida and to promote the production of high-value chemicals from its lignocellulose, we wanted to link genotypic, phenotypic, and chemotypic features of selected Sida accessions. Specifically, we aimed at answering the following questions: i) is geographic distance of Sida accessions reflected in the genetic distance (genotype) and plant phenotype, and ii) do genetically distant genotypes show differences in cell wall chemotype and recalcitrance? iii) Aiming at full valorization of Sida lignocellulose, can we identify suitable genotypes for an effective pretreatment via OrganoCat?

We report the collection of 16 Sida germplasms of North American and European origin and the construction of a phylogenetic tree using Single Nucleotide Polymorphisms (SNP) markers obtained by a genotyping by sequencing (GBS) approach. We identified genetically distant accessions and clonally propagated them by stem cuttings under controlled greenhouse conditions. Clonal plant material was used for phenotyping and in-depth wet chemical and spectroscopic cell wall analyses. We suggest that elucidation of a genotype-phenotype-chemotype nexus may lead to more specific strategies for targeted selection and cultivation of Sida accessions for biorefinery purposes.

## Materials and methods

### Establishing germplasm collection of *Sida hermaphrodita*

Seeds of Sida accessions were obtained from 16 different geographical origins. Three samples were collected in natural habitats in the USA, in the states of West Virginia and Kentucky, and others were obtained from botanical gardens, universities, and seed companies (**Supplementary Files Table S1**). The accessions were named according to the location of seedstock collection: SH1 (Kentucky), SH2 (West Virginia location 1), SH3 (West Virginia location 2), Jülich (J), Leipzig (L), Karlsruhe (K), Hohenheim (H), Hungary (Hu), Ukraine (UV and UF), Poland (P), Austria (A), Hungary-USA (Hu2), Düsseldorf (D) and Romania (R). One seed sample, (Je), was obtained commercially (Jellito Staudensamen GmbH, Schwarmstedt, Germany).

### GBS methodology and establishment of phylogenetic relations

DNA was isolated from young leaves of 192 Sida plants (from 2 to 18 seedlings per origin) using the DNeasy Plant Mini Kit (Qiagen, Hilden, Germany). Genotyping-by-sequencing (GBS) including SNP calling was performed commercially by the company LGC (Berlin, Germany). About 1.5 Mio read pairs (150 bp, Illumina NextSeq 500 V2) per sample were obtained. A surrogate reference genome was established by clustering all quality trimmed reads with CD-HIT-EST, allowing up to a 5% difference. Reads of every single plant were aligned to the reference clusters using Bowtie2 version 2.2.3, and variant discovery was performed with Freebayes v1.0.2-16. A total number of discovered SNPs across all samples consisted of 56,976 loci. A subset of 751 most reliable SNPs with allele frequency at or above 10% and min. read count of 8 in 128 samples was used in the genetic diversity study. Plant genotypes with more than 50% of missing alleles were discarded and a set of 174 genotypes of good sequence quality was analyzed further (**Supplementary Files Table S1**). The data presented in the study are deposited in the NCBI BioProject repository, accession number PRJNA971756.

Principal Component Analysis (PCA) was performed for 174 genotypes and 751 SNPs using R package SNPRelate (Zheng et al., 2012). Results were visualized using the matplotlib graphic package (Hunter, 2007). SVDQuartets (Singular Value Decomposition Scores for Species Quartets) appropriate for concatenated SNP datasets (Chifman and Kubatko, 2014) was used to infer relationships among quartets of taxa under the coalescent model. This program was implemented in the test version of PAUP* (Swofford, 2002). All possible quartets for each site in the alignment were estimated and combined with the species tree with the Quartet FM method (Reaz et al., 2014) implemented in PAUP*. The species-level phylogeny was estimated with all possible quartets, and 100 bootstrap replicates were performed to access the reliability of the tree topology. The resulting bootstrap consensus tree was visualized and edited with the software FigTree (Rambaut, 2012).

### Design of greenhouse experiments and phenotyping

**Figure 1:**
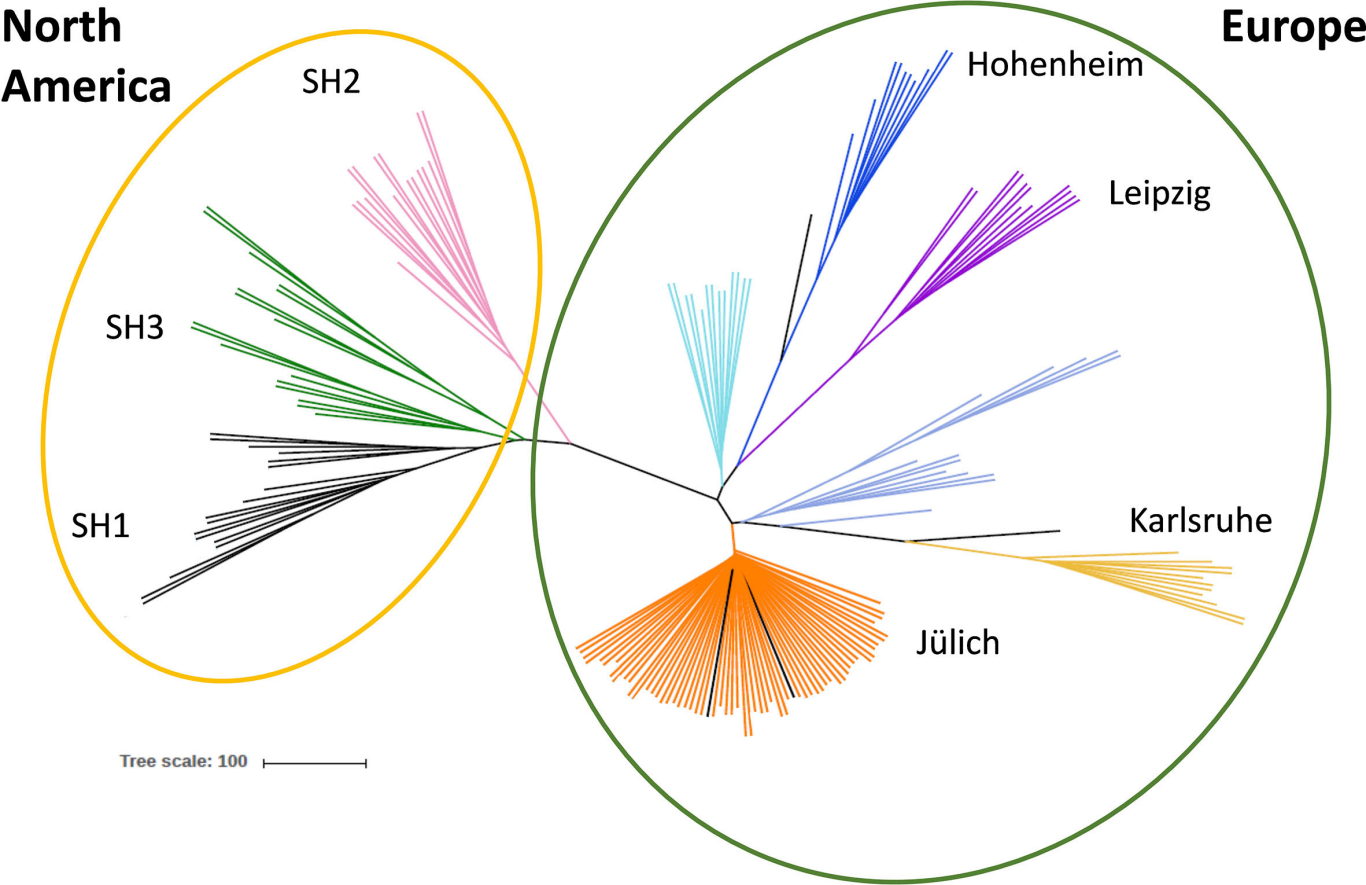
Genetic diversity of different Sida accessions. Unrooted coalescence tree obtained using the program SVDQuartets. The scale bar indicates the genetic distance. Genotypes collected in North America are shown in the yellow circle, European origins in the green circle. Color code indicates the accessions´ origins: black: SH1, green: SH3, pink: SH2, pale turquoise: Hu, dark blue: H, purple: L, violet blue: UV and UF, yellow: K, orange: Jül, Ju-A, P, US, D and J.

**Figure 2:**
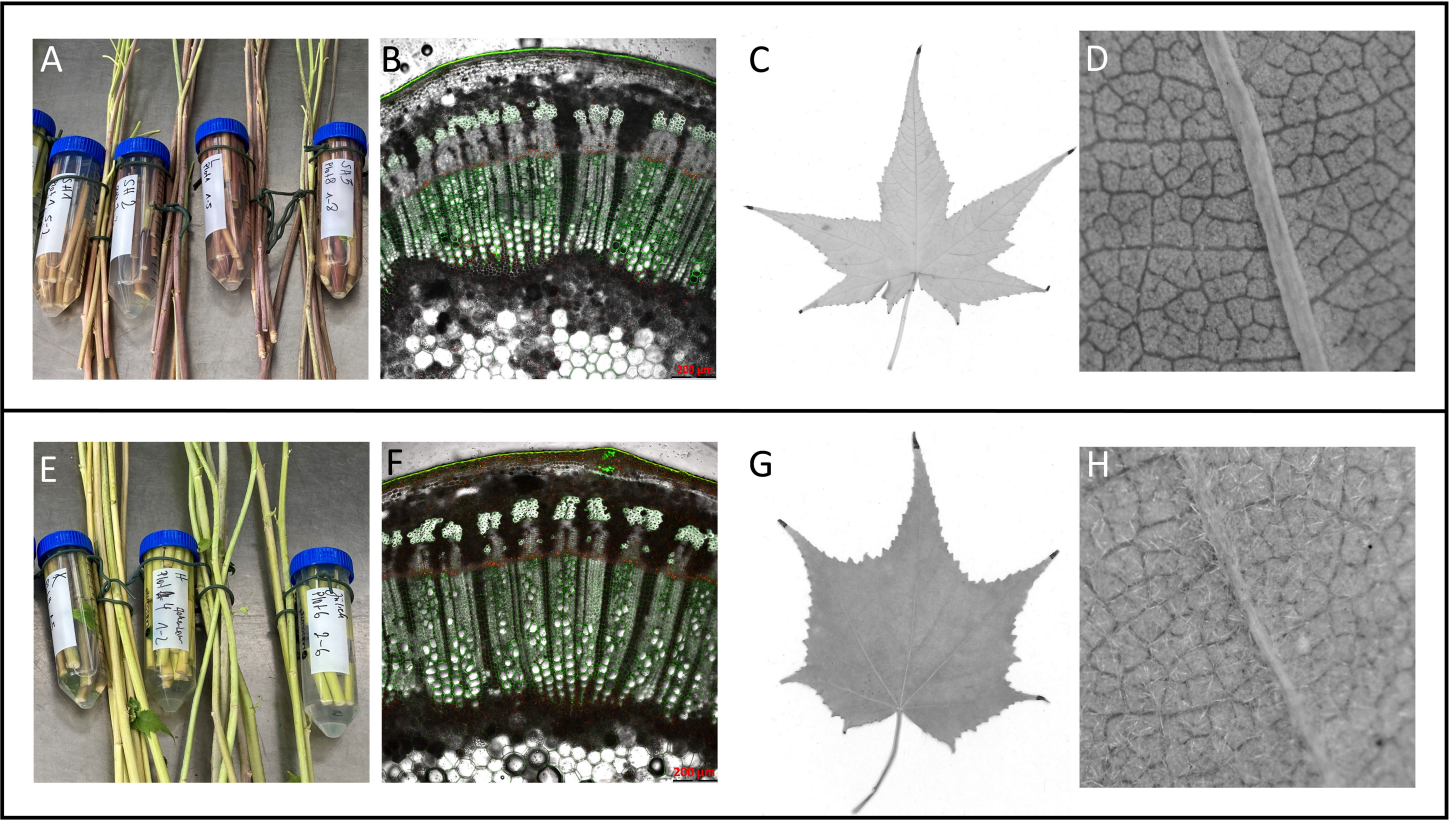
Grouping of Sida accessions according to phenotypic traits into a red-stemmed group **(A–D)** and a green-stemmed group **(E–H)**. **(A, E)** stem color at harvest, **(B, F)** stem cross-section, **(C, G)** typical leaf shape, **(D, H)**: presence of hair on abaxial leaf surface.

Seven genetically distinct Sida accessions were selected for further analysis based on the GBS results (see **Figures 1**, **2**). The following provenances were selected: the three accessions collected in the USA, i.e. SH1 (Kentucky), SH2 (West Virginia location 1), SH3 (West Virginia location 2), as well as Hohenheim (H), Jülich (J), Karlsruhe (K), and Leipzig (L). A specific mother plant was identified from each accession based on the number of shoots that could serve as material for stem cuttings, thus ensuring the highest possible number of daughter plants. Between 10 and 15 clones per accession were produced by propagation through stem cuttings in March 2021. For this purpose, stem cuttings with two to three nodes were rooted in a well-watered, nutrient-poor substrate (Einheitserde Werkverband e.V., Sinntal-Altengronau, Germany) in deep root trainer trays (HerkuPlast, Ering, Germany), covered with a translucent plastic hood, and sprayed daily with tap water for the first few weeks. Roots developed within the first two weeks, and after a total of four weeks, during which a good root system developed, the plants were transplanted into a nutrient-rich substrate (Lignostrat Dachgarten extensive, HAWITA, Vechta, Germany) to ensure abundant shoot growth. The pots were kept in greenhouse conditions to ensure identical growth. In September 2021, stem material was harvested, leaves and flowers were removed, and shoot material was dried at 65°C for cell wall analyses. The cut-back Sida plants were kept dormant over winter. In March 2022, plants were repotted into fresh nutrient-rich substrate and left to grow for subsequent phenotyping of stems and leaves.

To describe phenotypic traits, a total of 35 plants belonging to the 7 accessions of Sida clones grown in the greenhouse were visually evaluated on August 5, 2022. Phenotypic traits that were monitored were leaf shape, leaf pubescence, and stem color. Leaves were collected from each stem, dried, and pressed, and leaf shape was documented photographically. Pubescence was haptically scored as absent or present and leaves were observed under a binocular (Leica Microsystems, Wetzlar, Germany). For microscopic analyses, segments with identical diameters of 0.8 cm were selected and stored in 70% ethanol (pH ~ 5.9). Transverse sections of 25 µm thickness were prepared using Core-Microtome (Gärtner and Nievergelt, 2010). Microphotographs were obtained with Leica SP8 confocal microscope (Leica Microsystems, Wetzlar, Germany) using hardware focusing. For the imaging, HCX PL APO CS 10×/0.40 DRY objective was used. Autofluorescence was excited from a 488 nm 20 mW OPSL at 20% laser power. The reflected signal was collected at 500 nm – 570 nm and 680 nm – 750 nm to visualize flavonoids and chlorophyll respectively (Donaldson, 2020). Acquisition was achieved using PMT detectors operated with Leica LasX 2.0.1 software. Histogram correction was performed in all images identically using the Las X software.

### Cell wall chemotyping

Seven Sida accession were chemotyped using wet chemistry-based analysis of lignocellulose as previously described in Weidener et al. (2020). Briefly, Alcohol Soluble Compounds (ASC) were extracted by washing the biomass with 70% (v/v) ethanol and three times with a 1:1 (v/v) solution of chloroform:methanol, followed by one washing with acetone. This procedure resulted in the so-called Alcohol Insoluble Residues (AIR) biomass. De-starched biomass (dAIR) from the seven selected Sida accession was prepared by enzymatic digestion with amylglucosidase and α-amylase to remove starch and used for all further analysis. Lignin was determined as acetyl bromide soluble lignin (ABSL) and crystalline cellulose content by the Updegraff method as described by Foster et al. (2010a); Foster et al. (2010b). TFA hydrolysis by high-performance anion-exchange chromatography with pulsed amperometric detection (HPAEC-PAD), monosaccharide composition of the matrix polysaccharides in the AIR was determined, as described in Damm et al. (2017a). The total acetate content (acetyl groups) was determined using an acetic acid kit (Megazyme, Wicklow, Ireland), as previously described (Grande et al., 2019).

Reactions for saccharification tests were conducted in triplicates. Hydrolysis of lignocellulose raw material was carried out in an Eppendorf ThermoMixer Comfort using 1.5 mL Eppendorf vials. For each reaction, 20 mg material and 10 μL Accellerase^®^ 1500 (60 FPU mL−1 and 82 CBU mL−1, Genencor, The Netherlands) were dissolved in 1 mL of citrate buffer (pH = 4.5) and shaken at 50°C for 0, 1 and 72h. The samples were then heated to 90°C for 10 min to inactivate the enzymes. The glucose concentration was determined photometrically using a glucose (Hexokinase) assay kit obtained from Sigma-Aldrich. Glucose yields were calculated based on cellulose content as determined in the lignocellulose compositional analysis.

### OrganoCat pretreatment

Based on results on cellulose-to-lignin ratio and saccharification efficiency, SH1 and Hohenheim were chosen for OrganoCat analyses. Material from three clones from each of the two Sida accessions was pooled and AIR biomass was subjected to the standard OrganoCat fractionation (100 g/L biomass loading; 1:1, v/v, aqueous:organic phase) that has been established in previous studies (Grande et al., 2015). A microscale OrganoCat system was used in 5 replicates. Glass vials of 4 mL (CS-Chromatographie Service, Germany) were loaded with 100 mg biomass. One mL of 0.1M oxalic acid and 1 mL of 2-methyltetrahydrofuran (2-MTHF) and a micromagnetic stir bar (Length 6 mm, Carl Roth, Germany) were added. Then, the glass vials were tightly closed with a closed top-silicone septum screw polypropylene cap (CS-Chromatographie Service, Germany). An array of 10 vials per batch were placed in an oil bath at two different OrganoCat conditions (125°C and 140°C, respectively, for 3 h reaction time) and a rotational speed of 550 rpm. After the reaction took place, the vials were cooled down at room temperature. For better phase separation, the reaction products were transferred to an Eppendorf tube and centrifuged for 7 min at 14000 rpm. Then, 500 µl of the organic phase (lignin in 2-MTHF) was evaporated in a rotary evaporator and lignin was isolated. The rest of the organic phase was properly disposed of. The supernatant from the aqueous phase was centrifuged twice for 5 min at 14000 rpm and stored at -4°C for further analysis. The remaining pulp was washed with distilled water until neutral pH and left drying at 65°C until constant weight.

### Gravimetric determination of lignin and pulp yields

To determine lignin yield, a representative sample of 0.5 mL of the organic phase was collected and the resulting “Extracted lignin wt.” was extrapolated to the complete organic phase. Given that a minor fraction of 2-MTHF may be dissolved in the aqueous phase and only 0.5 mL were taken from the complete organic phase.


$$Lignin\;yield\;\left(\text{\% }original\;weight\right)=\frac{Extracted\;lignin\;wt.\;\left(mg\right)}{Initial\;biomass\;wt.\;\left(mg\right)}\times 1.92\times 100$$

For pulp yield, the ratio of dried pulp and initial biomass weight was calculated.


$$Pulp\;yield\;\left(\text{\% }orig.wt.\right)=\frac{Final\;pulp\;wt.\;\left(mg\right)}{Initial\;biomass\;wt.\;\left(mg\right)}\times 100$$

### Pulp analysis

The wet-chemical analysis was conducted in the same way as described for the cell wall chemotyping. Instead of using dAIR, the analysis started with pulp material and CrC, ABSL, Acetyl groups were determined. Residual non-cellulosic polysaccharides were hydrolyzed with TFA and resulting monosaccharides were quantified by HPAEC-PAD.

### Data analyses and statistical evaluation

A Principal Component Analysis (PCA) was conducted using the obtained biochemical parameters (**Supplementary Files Tables S2, S3**). For this, data were normalized by minimum-maximum scaling. Due to low availability of observations per variable, Gaussian distribution was not assumed. PCA was built based on variable maximization rotation method (Varimax with Kaiser) to redistribute cross loadings. Number of factors was selected based on eigenvalues greater than 1. After PCA was made, hierarchical clustering was performed, using factor loadings of Component 1 and 2, as they represent most of the variance (79.0%). A dendrogram was generated by Euclidian distances to identify clusters, which were chosen based on a 10% similarity coefficient of average linkage between groups. Pearson correlations between different parameters were made. Since normality could not be assumed for the dataset, Mann Whitney tests were performed for determining significant differences (p<0.05) between ecotypes and conditions. The statistical analyses were made by SPSS IBM SPSS Statistics v25.0 (2017).

## Results

### Genetic variation of Sida genotype collection

To analyze genetic diversity DNA samples of all 16 accession (in total 192) were sent for genotyping-by-sequencing (GBS) analysis (LGC, Berlin, Germany). A total number of discovered Single Nucleotide Polymorphisms (SNPs) consisted of 56,976 loci and the 751 most reliable of them were used in the genetic diversity study. After quality control, 174 plant samples could be evaluated in detail. Principal Component and phylogenetic analyses revealed that the American genotypes collected in three natural habitats are related to each other [shown in the yellow circle in **Figure 1**: SH1 (black), SH3 (green) and SH2 (pink)], and separated from the plants collected in Europe (green circle). Interestingly, the plants provided by different botanical gardens in Germany [Hohenheim (dark blue), Karlsruhe (yellow), and Leipzig, (purple)] are not closely related and form separated clusters. Six European origins (among them Jülich) formed one single cluster (orange) and, thus, could not be further distinguished (**Figure 1**). Based on the conducted analysis, individuals from seven genetically distinct accessions were selected for further study.

### Phenotyping of selected Sida accessions

Phenotypic characteristics of at least three clonal plants per accession growing under greenhouse conditions revealed that the morphological traits leaf form, hairiness, and stem color varied between the origins. Overall, two phenotypic groups could be determined: the American accessions SH1, SH2, and SH3 and Leipzig grouped while the remaining European accessions Karlsruhe, Jülich, and Hohenheim, formed a second group. This dichotomy was obvious in leaf shape, leaf hairiness, and stem color. The first group had reddish to dark reddish stems while the second group had yellowish-green stems. Microscopic analyses of stem cross-sections showed that the two groups differed in the signal emitted by the cuticula. In the red stemmed group, a bright green signal was observed while the green-stemmed group showed an orange-yellow signal. Xylem and phloem anatomy showed no histological differences under the selected conditions. This dichotomy corresponds to differences in leaf shape and hairiness. Sida leaves are arranged alternately along the stems, have a cordate base, are palmate with serrate margins, and have five to seven lobes, the middle lobe being the longest. Leaf surfaces were glabrous in the first group (SH1, SH2, SH3, and Leipzig, all red-stemmed) and densely hairy the second, green-stemmed group (Hohenheim, Jülich, Karlsruhe). Also, in leaves of the first group the cordate area was narrower while the lobes were longer. Group 2 had broader leaves with shorter lobes (**Figure 2**).

### Cell wall characterization

**Figure 3:**
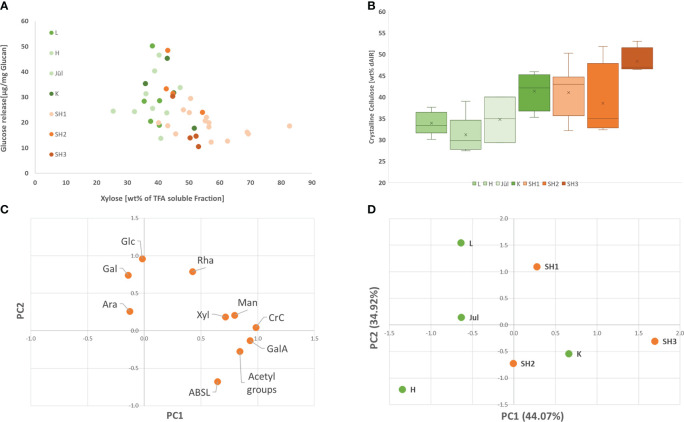
**(A)** Glucose release rate by saccharification with Accellerase^®^ 1500 (Genencore, NL) in respect to Xylose content for all analyzed clones from the seven selected accessions; **(B)** Crystalline Cellulose content in the seven selected accessions (Replicates of the different accessions: L: 5; H: 6; Jül: 3; K: 4; SH1: 15; SH2: 4; SH3: 4) **(C)**. Loading plot of the contribution of each analyzed variable for the prinicpal components PC 1 and 2: **(D)** principal component 1 and 2 for the seven analyzed accessions.

We analyzed crystalline cellulose content (CrC), acetyl groups, acetyl-bromide lignin (AcBr lignin) from dAIR, and total sugars from TFA fraction from the Sida biomasses (**Table 1** and **Supplementary Files Table S2**). Within the seven different accessions, the content of crystalline cellulose (CrC) varied between 31.1% and 48.3%. Hohenheim had the lowest CrC content while the highest value was found for SH3. Generally, the American accession exhibited higher CrC content than the European, Karlsruhe (41.4%) being an exception (**Figure 3B**). Acetyl groups varied between 2.9% and 5.4%, with the lowest content found in Leipzig and the highest in SH3. The measured lignin ranged between 15.4% (Leipzig) and 21.5% (SH3). The sugars solubilized by TFA represent hemicelluloses and pectic polysaccharides. Within this fraction, xylose is the most prominent non-cellulosic sugar, accounting for up to 56.1% of the TFA hydrolysate. With up to 11.1%, galacturonic acid, a major building block of pectin, is the second most abundant sugar in this fraction. Overall, these findings are in line with our previous studies (Damm et al., 2017b; Weidener et al., 2020). The recalcitrance of the Sida material was analyzed using a standard saccharification assay, in which the material was first digested with Accelerase^®^ 1500 for 72 h without pretreatment and the released glucose was measured (**Figure 3A**). The released glucose varied between 17.44% (SH3) and 34.39% (SH2).

**Table 1:** Overview of variance of measured cell wall features of the seven analyzed Sida accessions.

	µg/mg dAIR			TFA Soluble Fraction [wt%]							µg Glc/mg Glucan
	Acetic Acid	Cellulose	Lignin	Rhamnose	Arabinose	Galactose	Glucose	Xylose	Mannose	Gal. Acid	Saccharification
Mean	44.8	382.9	184.4	3	6.1	8.1	5.3	62	2.5	13	12.2
SD	9.7	65.2	18	0.6	0.6	1	1.3	2.9	0.6	1.4	1.2
Var	94.4	4255.9	323.4	0.4	0.4	1	1.8	8.2	0.4	1.9	1.4
Min	29.2	311.6	155.4	2	5.3	6.9	3.8	56.7	1.7	10.4	10.7
Max	54.6	483.3	211.3	3.7	7.2	9.6	7.7	64.9	3.5	14.3	13.7

Acetic acid, cellulose and lignin refer to the dAIR weight, sugar monomers refer to the TFA Soluble Fraction in dAIR, glucose per glucan (determined as glucose after Seaman hydrolysis and TFA-hydrolysate) is liberated by saccharification of the plant material with Accellerase® 1500 (Genencore, NL).

Looking at all analyzed clones of the seven accessions (**Supplementary Files Table S4**), crystalline cellulose and xylose content exhibited a low negative correlation with saccharification (Pearson -0.41 and -0.46) whereas lignin content did not exhibit a clear correlation with the saccharification efficiency within in the analyzed set. In order to identify contrasting accessions for the subsequent OrganoCat pretreatment and fractionation, a principal component analysis was conducted using the data from cell wall chemotyping (**Figures 3C, D**; **Supplementary Files Table S3**). The first two components PC1 and PC2 already explained 79.0% of the variance in this set (**Supplementary Files S6**). In PC1, acetic acid and crystalline cellulose were the dominant contributors, followed by galacturonic acid and xylose. In PC2, galactose and glucose, rhamnose contributed most (**Supplementary Files S7**). The saccharification appeared only in PC3, together with arabinose and PC3 explained 16.6% of the variance within the set.

### OrganoCat pretreatment and fractionation

To investigate how cell wall material of different Sidas performs under OrganoCat pretreatment and fractionation and how this improves the saccharification, two contrasting accessions were selected based on their classification in the principal component analysis.

Hohenheim had a low cellulose to lignin ratio but a high saccharification efficiency, whereas SH1 had a low cellulose to lignin ratio and a low saccharification efficiency (**Supplementary File Table S2**). Pretreatment was performed as recently described (Martinez Diaz et al., 2023) using the alcohol insoluble residue (AIR) to investigate the effects on the Sida cell walls.

**Figure 4:**
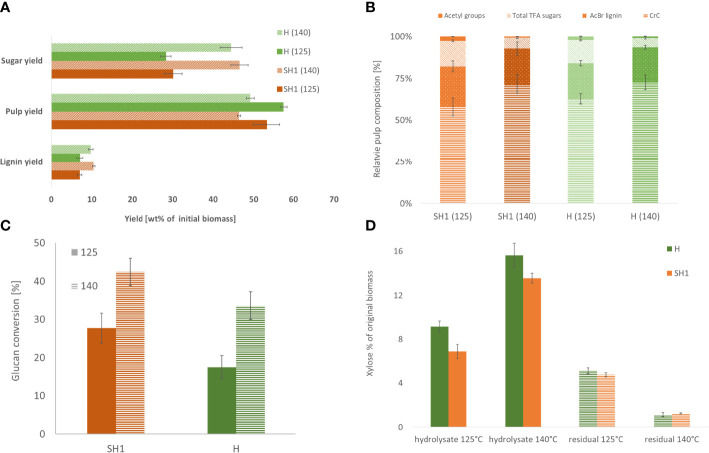
**(A)** Product yields of SH1 and Hohenheim OrganoCat processing at two different severities **(B)**. Relative pulp composition of SH1 and H derived from pretreatment at 125°C and 140°C **(C)**. Glucan (determined as glucose after Seaman hydrolysis and TFA-hydrolysate) conversion of OrganoCat pulp after 72h digestion with Accelerase. **(D)** Xylan hydrolysis by OrganoCat. Xylose in the hydrolysate fraction and residual xylose in the pulp (wt% in respect to initial biomass).

Comparing the two genotypes (**Supplementary File Table S8**), significant differences in pulp yield were observed both between the genotypes and at both severities (125°C, p=0.016; 140°C, p=0.008), while lignin yield differed only between the severities (p=0.008) (**Figure 4A**). There is no significant difference in composition between the pulp of the two accessions, except for CrC content under low severity (P=0.016, **Figure 4B**). The saccharification differs only at high severity between SH1 and Hohenheim, with SH1 exhibiting higher relative Glc release rate than H (**Figure 4C**).

During OrganoCat, mainly the non-cellulosic polysaccharides are hydrolyzed to monosaccharides. Glucuronoxylan is the main hemicellulose in Sida cell walls (Damm et al., 2017a), therefore xylose is also the main monosaccharide in the aqueous fraction (**Supplementary Files Table S9**). Hohenheim yielded more xylose than SH1 under both conditions. The pulp of the two accessions did not differ in residual xylose (**Figure 4D**).

## Discussion

Here we show that the geographical distance of Sida accessions is, in part, reflected in GBS data on Sida phylogeny. Selected accessions show distinct cell wall compositions and differences in recalcitrance towards OrganoCat pretreatment, suggesting that there may be accessions suitable for different processing technologies.

### *Sida hermaphrodita* phytogeography and genetic variability

We were able to collect Sida seed material from three natural reservoirs in West Virginia and Kentucky, USA. In their natural habitat Sida grows near river banks, wetlands, or floodplains (Borkowska and Molas, 2012), but also proliferates on roadsides, abandoned parking lots, and next to railway tracks (Groffman, 2020). Spooner et al. (1985) reported large populations in West Virginia and Ohio, and isolated populations in Kentucky, Michigan, and Indiana, however, Sida is becoming less widely spread and is even regarded as an endangered species, possibly due to continuous land-use changes (Thomas, 1979; Bickerton, 2011). Furthermore, recently Weakley et al. (2017) suggested reclassifying *Sida hermpahrodita* as *Ripariosida hermaphrodita* due to the phylogenetic, biogeographic, and morphologic isolation of *S. hermaphrodita* from other species of the genus, supported by molecular studies using nrDNA ITS sequence data (Aguilar et al., 2003; Tate et al., 2005). Thus, *S. hermpahrodita/R. hermaphrodita* is now regarded as a monotypic, isolated, North American temperate genus that is not congeneric with other members of the genus Sida (Weakley et al., 2017). These developments make the description, documentation, and conservation of its gene pool even more important. Our GBS results separated the European from the American accessions. Interestingly, the plants provided by different botanical gardens in Germany (Hohenheim, Karlsruhe, and Leipzig) are not closely related and form separated clusters suggesting bottleneck effects and subsequent inbreeding, while the six other European sources cluster together (Jülich, Jellito, Poland, Austria, Düsseldorf, and Hungary-USA), probably due to recent or regular exchange of seed material. Based on these results, we selected seven genetically distant accessions from the USA and Europe for further description of phenotypic differences and cell wall analysis.

### Phenotypic differences between Sida accessions

The seven selected Sida accessions were phenotypically divided into a red-stemmed and a green-stemmed cluster. In many vascular plants, stems are pigmented red while their leaves are green. Mostly, anthocyanins are the cause of the red coloration, although other pigments such as betalains and minor pigments, or other cell wall components like lignin may be involved (Davies, 2004). The pigments can be found in the epidermis and sub-epidermal cell layers (Wheldale, 1916; Nozzolillo and McNeill, 1985). In the Sida stem cross-section, the epidermis, collenchyma, phloem, cambial layer, and xylem can be easily identified, however, no typical anthocyanin-filled vacuoles could be observed. The difference in coloration between the two groups seems to be related to the thick unstructured cuticula layer on the epidermis that emits a yellow-greenish signal in the green-stemmed accessions and a bright green signal in the red-stemmed plants. This could be related to flavonoid composition. Indeed, members of the Malvaceae family are proliferate producers of biologically active chemical constituents, among them flavonoids, polyphenols, vitamins, terpenes, and tannins (Sharifi-Rad et al, 2020). Specifically, rutin, a flavonol glycoside, has been identified and extracted from epigeal *Sida hermaphrodita* parts (Bandyukova and Ligai, 1987). Rutin has been shown to function in e.g. UV protection, cold or desiccation tolerance (Suzuki et al., 2005). The biosynthesis of flavonoids, including rutin and anthocyanin, occurs via the phenylpropanoid pathway, which also gives rise to lignin (Zhang et al., 2021). In poplar, the manipulation of anthocyanin and lignin co-regulation has resulted in plants with reduced lignin and anthocyanin content and enhanced saccharification efficiency (Kim et al., 2021). However, the co-regulation network is complex and can vary depending on the plant species and environmental conditions. Further research is needed to clarify the identity of the compound(s) that cause the red stems of Sida plants and the impact on their processability and saccharification efficiency. Corresponding to the stem color, the accessions vary in leaf shape and hairiness. The red-stemmed group is associated with glabrous, narrowly cordate, deeply dissected, long-lobed leaves while the green-stemmed plants´ leaves are broad and hairy with short, pointy lobes. Differences in overall leaf shapes and leaf dissection within a species or even on individual plants are widespread. A strong correspondence exists between leaf form and climate within certain species, e.g. oak and *Acer* (Royer et al., 2008; Royer et al., 2009). Leaf pubescence as a trait has similar connotations. Increased leaf hairiness is the most commonly observed leaf morphological change in water-limited environments because it reduces water loss (Ehleringer and Björkman, 1978; Ehleringer and Mooney, 1978; Picotte et al., 2007). Recently, Pshenichnikova et al. (2019) identified density and length of leaf pubescence as important factors of diversity in the response to water deficiency among wheat genotypes. Interestingly, Franzaring et al. (2014) identified stellate trichomes on both ab- and adaxial leaf surfaces of Sida varieties raised from Polish and German seed supplies, however, the geographical origin of the accessions was unknown. In contrast, Weakley et al. (2017) describe Sida/*Ripariosida* collected in the natural habitat as hairless, corroborating our observations on the Sida accessions collected from the USA (hairless) and Europe (hairy). Overall, these observations indicate that the Sida accessions might in fact be adapted to different temperature or moisture availabilities, or even climatic conditions; a factor that should be considered when comparing studies or selecting accessions for the locally available soil fertility, water supply, and intended management intensity. Furthermore, such factors are closely related to cell wall formation (Tenhaken, 2015), making Sida a suitable crop for studying the effects of environmental conditions on lignocellulose, especially because Sida reaches the final stage of growth within one year.

### Sida cell wall chemotypic variance and recalcitrance

In the past, we created a reference profile for Sida cell walls (Damm et al., 2017a; Weidener et al., 2020) based on the Jülich accession. Generally, the Sida cell wall data generated here are in line with our previous findings. However, there are considerable differences between the accessions, especially in CrC and ABSL, which can affect subsequent valorization strategies (Yao et al., 2018; Yoo et al., 2020). In contrast, the variation in enzymatic saccharification between the accessions is comparably small. In all accessions, the xylose content had a negative effect on saccharification. Most of the xylose forms a glucuronoxylan backbone in Sida (Damm et al, 2017a), and xylan has been described as forming a physical barrier to the enzymatic hydrolysis of cellulose by blocking enzyme access to the cellulose surface (Qing and Wyman, 2011). Indeed, an initial pretreatment to reduce some of the limiting factors generally increases the glucose release rate derived by cellulases.

The data on chemotypic differences between Sida accessions suggest that careful selection of a specific accession for a particular valorization strategy could bring benefits. For example, SH1 with a high cellulose-to-lignin ratio might require less pretreatment and thus a less energy-intensive process to provide glucose as a substrate for subsequent fermentation. To test what impact the differences in cell wall characteristics would have on a biorefinery process, we used the OrganoCat pretreatment and fractionation system, which has been shown to be able to process Sida biomass (Damm et al., 2017b). Although the OrganoCat technology may produce technical lignins, here we focused on the polysaccharides in the pulp and hemicellulose fraction. The pretreatment of Hohenheim and SH1 resulted in a higher saccharification efficiency for both accessions. In both setups SH1 exhibited higher glucan conversion rates than Hohenheim, which, interestingly, cannot be easily assigned to any compositional feature we obtained in the pulp characterization. Overall, even though the two selected varieties (H and SH1) did not show strong differences following OrganoCat pretreatment these are, however, statistically significant, proving that the OrganoCat process can serve as an analytical technique to elucidate factors involved in cell wall recalcitrance. 

## Conclusion and outlook

The complex chemical nature of plant cell walls and improved processes for fractionation and conversion of biomass components open up opportunities for the development of high-value products that go beyond the use of plant biomass for energy production. We show here that there is indeed unexplored diversity within individual accessions of the species Sida, evident in genotype, phenotype and cell wall composition. To further elucidate the composition of Sida cell walls, the analysis of the ratio of syringyl (S) and guaiacyl (G) subunits of lignin yield will be of great importance, as lignin is a crucial factor for saccharification in many plant species. In addition, field studies need to be carried out to obtain biomass yields data of the genotypes so that correlations between yield and composition can be established. In future, this would make it possible to establish economic links between composition, yield and the conversion process.

Perennial plants are considered cost effective and low maintenance due to their life strategy, which is based on securing nutrients in underground storage organs over winter while the above-ground biomass dies. The following year’s regrowth is then independent of additional fertilization. Also, many ecological benefits, like offering protective cover for animals or reducing water loss and erosion of soils depend on the completion of this life strategy. The nutrient export associated with harvesting plants in the green state to ensure a certain biomass composition increases cultivation costs (Ruf and Emmerling, 2021). Thus, in the future we need to optimize lignocellulose use of such plants when harvested in the matured or dead state. The identification of stable traits of naturally dried biomass of different plant species or even genotypes of the same species would be an important goal on the way to a multipurpose use of perennial biomass plants such as Sida.

## Data availability statement

The original data used for this study are included in the article / **Supplementary Material**; further inquiries should be directed to the corresponding author. The sequencing data presented in this study are deposited in the NCBI BioProject repository, and available under the accession number PRJNA971756.

## Author contributions

SDS, PG, HK conceptualized the study, analyzed, visualized and interpreted the data. SDS wrote the first draft of the manuscript. JMD, JAM, BO, DD, PC, conducted wet chemical, histological and greenhouse experiments, and analyzed and interpreted the data. EP, LB and CW collected Sida seedstock, conducted genetic characterization and interpreted the phylogenetic data. HK, EP, NDJ and PG acquired funding. All authors contributed to the article and approved the submitted version.
