# Supplementary material for: Cell wall composition and biomass saccharification potential of Sida hermaphrodita differ between genetically distant accessions
Source: Front Plant Sci. 2023 Jun 29;14:1191249. doi: 10.3389/fpls.2023.1191249 (PMC10340120; doi:10.3389/fpls.2023.1191249)
Supplement: Supplementary file 1 [file DataSheet_1.docx]

Supplementary Material

Cell wall composition and biomass saccharification potential of Sida hermaphrodita differ between genetically distant accessions

Silvia D. Schrey, Jimena Martinez Diaz, Lukas Becker, Jane A. Mademann, Benedict Ohrem, Dagmar Drobietz, Pavel Chaloupsky, Nicolai D. Jablonowski, Christian Wever, Philipp M. Grande, Elena Pestsova, Holger Klose

*** Correspondence:**Corresponding Author
s.schrey@fz-juelich.de

**Table S1. Sida accessions from 16 different origins, their identities in the phylogenetic tree, their geographical origins and their contribution to phylogenetic analyses.**

| No | Identity | Identity (phyl. tree) | Origin | Country of origin | No. plants analysed by GBS | No. plants after GBS quality control | Provider / Accession number |
| --- | --- | --- | --- | --- | --- | --- | --- |
| 1 | SH1_P | 1P... | natural habitat, Kentucky | USA | 18 | 17 | Christian Wever / CW2016131 |
| 2 | SH2_P | 2P... | natural habitat, West Virginia | USA | 16 | 16 | Christian Wever / CW2016132 |
| 3 | SH3_P | 3P... | natural habitat, West Virginia | USA | 18 | 17 | Christian Wever / CW2016135 |
| 4 | SH_H | H... | Botanical Garden Hohenheim | Germany | 13 | 10 | XX-0-HOH-SYS-3993 |
| 5 | SH_L | L... | Botanical Garden Leipzig | Germany | 12 | 12 | XX-0-LZ-WNA-7-2013 |
| 6 | SH_UF | UF... | variety Fitoenergia | Ukraine | 13 | 8 | Plant Production Institute nd. A. V.Ya.Yuryev of the National Academy of Agrarian Science of Ukraine, Kharkiv |
| 7 | SH_UV | UV... | variety Virdjinia | Ukraine | 7 | 6 | Plant Production Institute nd. A. V.Ya.Yuryev of the National Academy of Agrarian Science of Ukraine, Kharkiv |
| 8 | SH_J | J... | Jelitto company | Germany | 13 | 13 | Jelitto perennial seeds |
| 9 | SH_Jü | Ju... S... | Forschungzentrum Jülich | Poland / Germany | 13 | 13 | Forschungzentrum Jülich, Field Daubenrath 2014 |
| 10 | SH_A | A... | Forschungzentrum Jülich | Austria | 13 | 13 | Forschungzentrum Jülich, N.D. Jablonowski |
| 11 | SH_Hu | Hu... | University of Debrecen | Hungary | 13 | 13 | Erika Kurucz (selection of Dr. Zoltán Kováts) |
| 12 | SH_HUS | US... | University of Debrecen | USA / Hungary | 13 | 13 | Erika Kurucz (USA, natural population 2009) |
| 13 | SH_K | K... | BG KIT, Karlsruhe | Germany | 12 | 11 | - |
| 14 | SH_P | P... | University of Warmia and Mazury in Olsztyn | Poland | 13 | 8 | Jacek Kwiatkowski |
| 15 | SH_R | R... | University of Târgu Mureș | Romania | 2 | 1 | - |
| 16 | SH_D | D... | Botanical Garden Düsseldorf | Germany | 3 | 3 | - |
| Total |  |  |  |  | 192 | 174 |  |

**Table S2. Mean values of Cell wall composition (chemotype) of the seven different Sida accessions.** Mean values of and standard deviation (stdev.) of crystalline cellulose (CrC), acetyl groups, acetyl bromide lignin (ABSL) and polysaccharides in TFA fraction, in % of the total TFA fraction; Leipzig (L): n=5; Hohenheim (H): n=6; Jülich (Jül): n=3; Karlsruhe (K): n=4; Kentucky (SH1): n=15; West Virginia location 1 (SH2): n=4; West Virginia location 2 (SH3): n=4.

| Accession |  | Acetyl groups | CrC | ABSL | Hemicellulose (total TFA) | Rhamnose | Arabinose | Galactose | Glucose | Xylose | Mannose | Gal. Acid | 72h Accellerase |
| --- | --- | --- | --- | --- | --- | --- | --- | --- | --- | --- | --- | --- | --- |
|  |  | wt% / dAIR | | | | µg / mg dAIR | | | | | | | wt% Glc / Glucan |
| L | mean | 2.92 | 33.88 | 15.54 | 18.03 | 2.40 | 4.25 | 6.47 | 5.18 | 38.33 | 1.79 | 9.18 | 29.39 |
|  | stdev. | 0.49 | 2.78 | 0.97 | 1.49 | 0.86 | 0.72 | 1.18 | 0.75 | 2.09 | 0.35 | 1.22 | 12.48 |
| H | mean | 3.71 | 31.16 | 19.75 | 15.00 | 1.10 | 2.99 | 4.04 | 3.10 | 36.51 | 1.45 | 7.06 | 31.09 |
|  | stdev. | 0.99 | 4.44 | 1.11 | 2.46 | 0.38 | 0.32 | 0.39 | 0.90 | 7.36 | 0.42 | 0.48 | 8.56 |
| Jül | mean | 3.98 | 34.73 | 17.94 | 18.26 | 2.29 | 4.95 | 6.09 | 3.99 | 40.78 | 1.95 | 8.42 | 26.01 |
|  | stdev. | 1.06 | 5.36 | 0.60 | 1.81 | 0.79 | 0.65 | 1.58 | 0.71 | 1.98 | 0.96 | 1.00 | 13.42 |
| K | mean | 5.47 | 41.35 | 19.03 | 19.17 | 2.33 | 4.47 | 5.52 | 3.13 | 43.87 | 1.83 | 10.74 | 32.60 |
|  | stdev. | 0.00 | 2.08 | 0.96 | 2.16 | 1.02 | 1.32 | 0.73 | 1.17 | 4.14 | 1.00 | 1.60 | 12.94 |
| SH1 | mean | 5.15 | 41.03 | 17.37 | 19.56 | 3.30 | 5.28 | 7.71 | 5.14 | 56.11 | 1.45 | 9.12 | 19.34 |
|  | stdev. | 0.65 | 5.72 | 3.17 | 2.53 | 1.26 | 1.46 | 2.03 | 1.99 | 11.18 | 1.09 | 5.14 | 4.62 |
| SH2 | mean | 4.72 | 38.53 | 18.18 | 19.35 | 1.78 | 4.37 | 6.08 | 2.74 | 46.19 | 1.37 | 10.01 | 34.39 |
|  | stdev. | 0.53 | 8.98 | 0.46 | 1.91 | 0.58 | 0.38 | 0.46 | 0.88 | 5.52 | 1.36 | 0.79 | 10.26 |
| SH3 | mean | 5.46 | 48.33 | 21.13 | 20.28 | 2.15 | 4.60 | 5.48 | 3.51 | 50.09 | 2.83 | 11.15 | 17.44 |
|  | stdev. | 0.46 | 3.12 | 1.62 | 1.33 | 1.09 | 0.83 | 0.53 | 1.51 | 3.81 | 1.22 | 1.76 | 8.88 |

**Table S3. Normalized data of cell wall characterization of untreated biomass for construction of PCA in SPSS.**

| Accession | Acetyl groups | CrC | ABSL | Hemicellulose (total TFA) | Rhamnose | Arabinose | Galactose | Glucose | Xylose | Mannose | Gal. Acid |
| --- | --- | --- | --- | --- | --- | --- | --- | --- | --- | --- | --- |
|  | wt%/ dAIR | | | | µg/mg dAIR | | | | | | |
| L | 0 | 0.26 | 0 | 0.86 | 0.58 | 0.22 | 0.52 | 1 | 0.03 | 0.42 | 0.3 |
| H | 0.28 | 0 | 0.55 | 0 | 0 | 0.54 | 0.13 | 0.1 | 0 | 0 | 0 |
| Jül | 0.42 | 0.13 | 0.43 | 0.04 | 0.53 | 0.73 | 0.36 | 0.51 | 0.17 | 0.51 | 0.03 |
| K | 0.91 | 0.64 | 0.63 | 1 | 0.49 | 0 | 0 | 0.12 | 0.36 | 0.44 | 0.71 |
| SH1 | 0.88 | 0.63 | 0.33 | 0.22 | 1 | 1 | 1 | 0.98 | 1 | 0.33 | 0.34 |
| SH2 | 0.71 | 0.5 | 0.47 | 0.9 | 0.28 | 0.31 | 0.36 | 0 | 0.48 | 0.21 | 0.59 |
| SH3 | 1 | 1 | 1 | 0.25 | 0.52 | 0.47 | 0.11 | 0.42 | 0.7 | 1 | 1 |

**Table S4. Cell wall composition (chemotype) of 41 clones of the seven different Sida accessions.** Mean values of the individual clones. Crystalline cellulose (CrC), acetyl groups, acetyl bromide lignin (ABSL) and polysaccharides in TFA fraction, in % of the total TFA fraction;

| No | Accession | Acetyl groups | CrC | ABSL | Hemicellulose (total TFA) | Rhamnose | Arabinose | Galactose | Glucose | Xylose | Mannose | Gal. Acid | 72h Accellerase |
| --- | --- | --- | --- | --- | --- | --- | --- | --- | --- | --- | --- | --- | --- |
|  |  | wt%/ dAIR | | | | µg/mg dAIR | | | | | | | wt% Glc/ Glucan |
| 1 | L | 2.52 | 37.61 | 14.39 | 17.18 | 1.10 | 4.43 | 5.46 | 5.95 | 37.51 | 1.93 | 8.04 | 20.60 |
| 2 | L | 2.55 | 30.11 | 14.69 | 19.79 | 3.28 | 4.95 | 8.01 | 5.68 | 40.30 | 1.51 | 10.46 | 18.97 |
| 3 | L | 2.81 | 33.35 | 16.24 | 19.48 | 2.90 | 4.79 | 7.23 | 5.52 | 40.39 | 1.80 | 10.43 | 28.70 |
| 4 | L | 3.00 | 33.07 | 16.61 | 17.10 | 2.69 | 3.88 | 6.44 | 4.46 | 35.40 | 2.30 | 8.97 | 28.45 |
| 5 | L | 3.73 | 35.25 | 15.75 | 16.58 | 2.01 | 3.18 | 5.21 | 4.31 | 38.06 | 1.40 | 8.01 | 50.26 |
| 6 | H | 2.74 | 28.24 | 19.01 | 14.71 | 0.85 | 3.14 | 3.96 | 2.76 | 35.96 | 1.52 | 6.97 | 31.38 |
| 7 | H | 5.22 | 39.00 | 18.57 | 13.63 | 1.32 | 2.58 | 4.14 | 2.79 | 32.28 | 0.99 | 7.02 | 24.35 |
| 8 | H | 3.42 | 27.43 | 20.04 | 15.86 | 0.81 | 3.19 | 3.63 | 2.92 | 40.21 | 1.58 | 7.13 | 46.65 |
| 9 | H | 4.63 | 33.06 | 20.61 | 16.16 | 1.71 | 2.95 | 4.69 | 3.53 | 38.15 | 2.04 | 7.51 | 25.69 |
| 10 | H | 2.89 | 31.37 | 21.37 | 18.45 | 0.71 | 3.41 | 4.18 | 4.63 | 47.08 | 1.65 | 7.53 | 33.83 |
| 11 | H | 3.36 | 27.86 | 18.88 | 11.20 | 1.19 | 2.68 | 3.65 | 1.98 | 25.34 | 0.95 | 6.22 | 24.62 |
| 12 | Jül | 3.34 | 34.91 | 17.30 | 20.35 | 3.12 | 5.68 | 7.86 | 4.81 | 42.75 | 3.05 | 9.05 | 23.86 |
| 13 | Jül | 5.20 | 40.01 | 18.50 | 17.26 | 2.21 | 4.41 | 5.61 | 3.55 | 38.78 | 1.24 | 8.94 | 40.37 |
| 14 | Jül | 3.39 | 29.28 | 18.01 | 17.17 | 1.54 | 4.76 | 4.81 | 3.62 | 40.81 | 1.56 | 7.26 | 13.80 |
| 15 | K | 5.47 | 35.27 | 18.01 | 16.08 | 2.16 | 3.95 | 5.44 | 2.05 | 35.82 | 0.87 | 10.01 | 35.38 |
| 16 | K | 5.47 | 45.89 | 20.49 | 22.00 | 3.46 | 4.29 | 5.25 | 4.25 | 51.71 | 2.04 | 11.50 | 17.86 |
| 17 | K | 5.47 | 43.17 | 19.33 | 18.56 | 1.01 | 3.59 | 4.98 | 2.78 | 45.02 | 2.70 | 9.52 | 31.78 |
| 18 | K | 5.47 | 41.06 | 18.30 | 20.05 | 2.69 | 6.07 | 6.41 | 3.45 | 42.92 | 1.72 | 11.92 | 45.37 |
| 19 | SH1 | 5.33 | 44.67 | 18.68 | 16.92 | 2.28 | 3.02 | 4.76 | 1.49 | 43.03 | 0.32 | 8.57 | 18.82 |
| 20 | SH1 | 4.58 | 44.59 | 21.06 | 23.31 | 1.94 | 3.40 | 7.45 | 3.63 | 57.17 | 1.53 | 12.28 | 12.40 |
| 21 | SH1 | 3.76 | 43.68 | 18.94 | 21.50 | 3.69 | 4.14 | 7.26 | 4.83 | 82.62 | 2.51 | 12.33 | 18.75 |
| 22 | SH1 | 5.60 | 41.90 | 13.79 | 23.22 | 5.22 | 6.41 | 9.53 | 7.74 | 68.83 | 2.86 | 17.08 | 16.32 |
| 23 | SH1 | 5.38 | 46.69 | 20.74 | 20.76 | 3.92 | 6.90 | 8.18 | 6.02 | 62.52 | 3.39 | 14.71 | 12.78 |
| 24 | SH1 | 4.79 | 32.70 | 15.27 | 21.88 | 2.40 | 5.61 | 6.60 | 3.90 | 50.50 | 0.88 | 12.15 | 29.56 |
| 25 | SH1 | 5.30 | 36.38 | 19.35 | 21.15 | 2.86 | 5.29 | 6.48 | 5.53 | 45.66 | 1.46 | 12.03 | 15.63 |
| 26 | SH1 | 5.43 | 44.94 | 16.52 | 18.23 | 2.84 | 3.71 | 4.85 | 4.10 | 69.28 | 2.33 | 12.07 | 15.59 |
| 27 | SH1 | 4.89 | 32.14 | 15.49 | 22.21 | 0.99 | 4.80 | 6.18 | 3.52 | 56.44 | 0.84 | 10.49 | 20.00 |
| 28 | SH1 | 5.35 | 42.96 | 23.15 | 18.99 | 2.35 | 4.21 | 6.70 | 4.64 | 56.46 | 1.12 | 12.38 | 18.31 |
| 29 | SH1 | 5.51 | 50.22 | 14.70 | 16.45 | 3.67 | 7.34 | 11.61 | 6.43 | 50.07 | 2.16 | 2.67 | 23.95 |
| 30 | SH1 | 5.67 | 43.90 | 13.81 | 17.15 | 5.30 | 5.96 | 8.80 | 8.34 | 55.61 | 2.21 | 2.82 | 22.18 |
| 31 | SH1 | 5.79 | 35.62 | 12.15 | 17.74 | 2.79 | 8.00 | 8.14 | 4.65 | 48.19 | 0.01 | 2.20 | 25.07 |
| 32 | SH1 | 3.90 | 32.20 | 17.09 | 16.97 | 4.72 | 5.22 | 7.63 | 3.59 | 40.05 | 0.21 | 2.23 | 19.94 |
| 33 | SH1 | 6.00 | 42.95 | 19.80 | 16.86 | 4.46 | 5.20 | 11.47 | 8.63 | 55.21 | 0.00 | 2.82 | 20.75 |
| 34 | SH2 | 5.20 | 35.78 | 18.41 | 17.32 | 1.19 | 3.96 | 5.97 | 2.13 | 42.57 | 0.19 | 8.92 | 33.37 |
| 35 | SH2 | 5.11 | 32.36 | 18.49 | 19.03 | 2.57 | 4.73 | 6.72 | 1.88 | 44.75 | 0.27 | 10.46 | 31.52 |
| 36 | SH2 | 4.10 | 51.83 | 18.32 | 21.94 | 1.72 | 4.14 | 5.99 | 3.26 | 54.36 | 2.08 | 10.70 | 24.12 |
| 37 | SH2 | 4.45 | 34.14 | 17.50 | 19.11 | 1.64 | 4.67 | 5.64 | 3.71 | 43.09 | 2.92 | 9.98 | 48.56 |
| 38 | SH3 | 5.88 | 53.00 | 20.32 | 19.37 | 3.04 | 4.52 | 5.14 | 5.41 | 53.10 | 4.56 | 11.94 | 10.59 |
| 39 | SH3 | 5.83 | 46.78 | 22.59 | 22.18 | 0.97 | 5.33 | 5.70 | 3.99 | 52.27 | 2.71 | 12.19 | 14.71 |
| 40 | SH3 | 5.15 | 46.44 | 22.36 | 20.26 | 3.12 | 5.09 | 6.13 | 2.64 | 44.65 | 2.35 | 11.97 | 30.49 |
| 41 | SH3 | 4.98 | 47.08 | 19.24 | 19.32 | 1.47 | 3.46 | 4.97 | 2.00 | 50.34 | 1.70 | 8.51 | 13.99 |

**Table S5. Pearson Correlation of Enzyamtic Saccharificaten (Glucose release / Glucan) with different cell wall features.**

|  |  |  |  |  |  |  |  |  |  |  |  |
| --- | --- | --- | --- | --- | --- | --- | --- | --- | --- | --- | --- |
|  | Acetyl groups | CrC | ABSL | Hemicellulose (total TFA) | Rhamnose | Arabinose | Galactose | Glucose | Xylose | Mannose | Gal. Acid |
|  | wt%/ dAIR | | | | µg/mg dAIR | | | | | | |
| all | -0.19 | -0.41 | -0.09 | -0.32 | -0.31 | -0.17 | -0.26 | -0.30 | -0.46 | -0.18 | -0.17 |

**Table S6. Scores of the first three Components for each accession**

| Accession | PC1 | PC2 | PC3 |
| --- | --- | --- | --- |
| L | -0.637 | 1.542 | -1.383 |
| Jul | -0.636 | 0.144 | 0.219 |
| H | -1.345 | -1.216 | 0.238 |
| K | 1.694 | -0.305 | -0.232 |
| SH1 | -0.011 | -0.725 | 0.143 |
| SH2 | 0.660 | -0.541 | -0.795 |
| SH3 | 0.275 | 1.101 | 1.810 |

**Table S7. Scores of the first three Components for each accession**

|  | PC1 | PC2 | PC3 |
| --- | --- | --- | --- |
| Acetyl groups | 0.841 | -0.276 | 0.399 |
| CrC | 0.984 | 0.044 | 0.065 |
| ABSL | 0.645 | -0.679 | 0.086 |
| Rha | 0.424 | 0.792 | 0.356 |
| Ara | -0.127 | 0.259 | 0.868 |
| Gal | -0.143 | 0.742 | 0.609 |
| Glc | -0.018 | 0.961 | 0.136 |
| Xyl | 0.714 | 0.187 | 0.645 |
| Man | 0.794 | 0.209 | -0.230 |
| GalA | 0.930 | -0.128 | -0.230 |
| Acetyl groups | 0.841 | -0.276 | 0.399 |

**Table S8. Wet-chemical composition of the pulp of Hohenheim and SH1 Accession after OrganoCat pretreatment.** Mean values and standard deviation of Crystalline cellulose (CrC), acetyl groups, acetyl bromide lignin (ABSL) and polysaccharides in TFA fraction, in % of the total TFA fraction; n =5.

|  |  |  |  | |  |  |  |  |  | |  | |  | |  |  |  | |  | |  | |  | |  |
| --- | --- | --- | --- | --- | --- | --- | --- | --- | --- | --- | --- | --- | --- | --- | --- | --- | --- | --- | --- | --- | --- | --- | --- | --- | --- |
| Accession | | conditions | Acetyl groups | | CrC | ABSL | Total TFA sugars | Pulp | Fuc | | Rha | | Ara | | Gal | Glc | Xyl | | Man | | Gal. Acid | | Glc. Acid | | Saccharification |
|  |  |  | [% sample biomass wt.] | | | | | | TFA fraction [% sample biomass wt.] | | | | | | | | | | | | | | | | (wt% Glc/ Glucan) |
| SH1 | mean | 125 °C, 3 h | 2.28 | 52.24 | | 21.97 | 13.81 | 53.2 | n.a. | 0.25 | | 0.01 | | 0.88 | | 2.14 | | 9.01 | | 0.77 | | 0.76 | | n.a. | 27.99 |
|  | stdev. |  | 0.10 | 4.83 | | 2.98 | 0.48 | 3.2 |  | 0.02 | | 0.00 | | 0.07 | | 0.18 | | 0.36 | | 0.03 | | 0.09 | |  | 5.02 |
|  | mean | 140 °C, 3 h | 0.72 | 69.74 | | 20.83 | 6.34 | 46.41 | n.a. | 0.07 | | n.a. | | 0.19 | | 2.78 | | 2.69 | | 0.42 | | 0.19 | | n.a. | 42.70 |
|  | stdev. |  | 0.05 | 5.72 | | 3.81 | 0.45 | 0.39 |  | 0.01 | | n.a. | | 0.02 | | 0.34 | | 0.10 | | 0.04 | | 0.02 | |  | 5.1 |
| H | mean | 125 °C, 3 h | 2.07 | 63.18 | | 21.53 | 14.01 | 57.49 | n.a. | 0.25 | | 0.03 | | 0.81 | | 2.27 | | 8.92 | | 0.83 | | 0.84 | | 0.06 | 17.53 |
|  | stdev. |  | 0.26 | 3.06 | | 1.57 | 0.50 | 0.92. |  | 0.01 | | 0.02 | | 0.04 | | 0.08 | | 0.47 | | 0.03 | | 0.05 | | 0.00 | 3.2 |
|  | mean | 140 °C, 3 h | 0.72 | 69.03 | | 19.99 | 5.36 | 49.17 | n.a. | 0.07 | | 0.01 | | 0.18 | | 2.21 | | 2.29 | | 0.43 | | 0.17 | | n.a. | 33.75 |
|  | stdev. |  | 0.15 | 4.29 | | 1.13 | 0.66 | 0.97 |  | 0.01 | | 0.00 | | 0.04 | | 0.35 | | 0.39 | | 0.02 | | 0.01 | |  | 4.5 |

**Table S9. Monosaccharide composition of the Hydrolysate fraction of Hohenheim and SH1 accession after OrganoCat pretreatment; n =3.**

| Accession | | conditions | Fucose | Rhamnose | Arabinose | Galactose | Glucose | Xylose | Mannose | Gal. Acid | Gluc. Acid |
| --- | --- | --- | --- | --- | --- | --- | --- | --- | --- | --- | --- |
|  |  |  | [wt% original biomass] | | | | | | | | |
| SH1 | mean | 125 °C, 3 h | 0.183 | 0.682 | 2.124 | 10.423 | 6.979 | 6.891 | 0.561 | 2.125 | 0.169 |
|  | stdev. |  | 0.019 | 0.096 | 0.183 | 0.607 | 0.493 | 0.649 | 0.090 | 0.205 | 0.015 |
|  | mean | 140 °C, 3 h | 0.186 | 1.988 | 1.992 | 13.056 | 10.613 | 13.559 | 1.343 | 3.319 | 0.455 |
|  | stdev. |  | 0.014 | 0.176 | 0.127 | 0.659 | 0.923 | 0.446 | 0.061 | 0.194 | 0.087 |
| H | mean | 125 °C, 3 h | 0.175 | 0.637 | 2.047 | 9.549 | 4.044 | 9.138 | 0.763 | 1.869 | 0.132 |
|  | stdev. |  | 0.009 | 0.048 | 0.087 | 0.684 | 0.284 | 0.508 | 0.050 | 0.097 | 0.006 |
|  | mean | 140 °C, 3 h | 0.198 | 1.836 | 2.091 | 12.398 | 7.089 | 15.637 | 1.753 | 3.027 | 0.414 |
|  | stdev. |  | 0.015 | 0.109 | 0.108 | 1.092 | 0.652 | 1.075 | 0.115 | 0.234 | 0.021 |
